# Implications of Glutathione Levels in the *Plasmodium berghei* Response to Chloroquine and Artemisinin

**DOI:** 10.1371/journal.pone.0128212

**Published:** 2015-05-26

**Authors:** Joel Vega-Rodríguez, Rebecca Pastrana-Mena, Keila N. Crespo-Lladó, José G. Ortiz, Iván Ferrer-Rodríguez, Adelfa E. Serrano

**Affiliations:** 1 Department of Microbiology and Medical Zoology, University of Puerto Rico, School of Medicine, San Juan, Puerto Rico; 2 Department of Pharmacology, University of Puerto Rico, School of Medicine, San Juan, Puerto Rico; Centro de Pesquisa Rene Rachou/Fundação Oswaldo Cruz (Fiocruz-Minas), BRAZIL

## Abstract

Malaria is one of the most devastating parasitic diseases worldwide. Plasmodium drug resistance remains a major challenge to malaria control and has led to the re-emergence of the disease. Chloroquine (CQ) and artemisinin (ART) are thought to exert their anti-malarial activity inducing cytotoxicity in the parasite by blocking heme degradation (for CQ) and increasing oxidative stress. Besides the contribution of the CQ resistance transporter (PfCRT) and the multidrug resistant gene (*pfmdr)*, CQ resistance has also been associated with increased parasite glutathione (GSH) levels. ART resistance was recently shown to be associated with mutations in the K13-propeller protein. To analyze the role of GSH levels in CQ and ART resistance, we generated transgenic *Plasmodium berghei *parasites either deficient in or overexpressing the *gamma-glutamylcysteine synthetase* gene (*pbggcs*) encoding the rate-limiting enzyme in GSH biosynthesis. These lines produce either lower (*pbggcs-ko*) or higher (*pbggcs-oe*) levels of GSH than wild type parasites. In addition, GSH levels were determined in *P*. *berghei* parasites resistant to CQ and mefloquine (MQ). Increased GSH levels were detected in both, CQ and MQ resistant parasites, when compared to the parental sensitive clone. Sensitivity to CQ and ART remained unaltered in both *pgggcs-ko* and *pbggcs-oe* parasites when tested in a 4 days drug suppressive assay. However, recrudescence assays after the parasites have been exposed to a sub-lethal dose of ART showed that parasites with low levels of GSH are more sensitive to ART treatment. These results suggest that GSH levels influence *Plasmodium berghei* response to ART treatment.

## Introduction

The development of drug resistance by *Plasmodium* parasites has become one of the major obstacles in the efforts to control malaria. *Plasmodium falciparum*, the deadliest and more severe malaria parasite, has developed resistance to the majority of the antimalarial drugs currently available [1]. Similarly, *Plasmodium vivax*, an important cause of malaria morbidity, has become resistant to chloroquine (CQ) and resistance has spread to almost all endemic countries [2]. Chloroquine (CQ), previously used as the first-line treatment and most cost-effective antimalarial, is currently ineffective in nearly all regions where malaria is endemic [1]. More alarming is the loss of sensitivity to artemisinin (ART), the current first-line treatment, which is emerging in the Thai-Cambodian border [3–5]. In addition, signs of *P*. *falciparum* ART resistance have been reported in Africa [6]. The development of drug resistance by malaria parasites poses a clear threat to recent efforts that have significantly reduced the burden of the disease.

Development of CQ resistance has been linked to the CQ resistance transporter (*pfcrt*) and the multidrug resistance analogue (*pfmdr1*) genes. However, glutathione (GSH)-mediated detoxification has been proposed to contribute to CQ resistance. A marked increase in GSH levels and the activity and expression of GSH-related enzymes has been reported in *P*. *berghei* and *P*. *falciparum* lines resistant to CQ [7–11]. In addition, a fraction of the toxic heme molecule produced during hemoglobin catabolism is detoxified by GSH, a process inhibited by CQ [8]. Therefore, increased GSH levels in the parasite might help overcome the CQ blockage of GSH-mediated heme degradation, resulting in an increased resistance to CQ [12].

The antimalarial activity of ART and its derivatives is proposed to be mediated by the iron-dependent generation of reactive oxygen species (ROS), which alters the redox balance of the parasite and consequently induces damage to cellular targets. ART reacts with hemin *in vitro* [13], and *in vivo*, the binding affinity to hemin correlates with the antiplasmodial activity of the drug [14]. In addition, the increased levels of intracellular ROS, and the antimalarial activity of ART require the uptake and degradation of hemoglobin by *Plasmodium* parasites [15]. Moreover, reduced GSH reacts and forms adducts with ART derived C-centered primary radicals [16], which might result in deprivation of GSH and consequently, an increase in intracellular ROS damage. As GSH is one of the parasite's main antioxidant systems, it is conceivable that increased levels of GSH could potentially detoxify the ROS-induced damage caused by ART treatment.

GSH is synthesized *de novo* by the sequential action of the rate-limiting enzyme gamma-glutamylcysteine synthetase (γ-GCS) and the GSH synthetase (GS) [17, 18]. Increased expression of the *pbggcs* mRNA was shown in *P*. *berghei* lines resistant to CQ and MQ [10]. Further evidence supporting a role for the *pbggcs* gene in CQ resistance comes from reports where the γ-GCS inhibitor L-buthionine sulfoximine (BSO) partially reverts the CQ resistance phenotype in *P*. *berghei* [7, 19]. In addition, CQ sensitive *P*. *falciparum* parasites are more susceptible to BSO treatment than CQ resistant parasites [9, 20]. These results support the association between increased GSH levels and CQ resistance in *Plasmodium*.

To further investigate the potential contribution of GSH to *Plasmodium* drug resistance, the development of genetically engineered *P*. *berghei* parasites overexpressing the *pbggcs* gene and displaying high levels of GSH is reported herein. We had previously disrupted the *pbggcs* gene, resulting in mutant parasites with significantly low levels of GSH [21]. Drug sensitivity responses were evaluated in mutants with the *pbggcs* silenced or overexpressed, as well as recrudescence and mice survival after treatment with an ART derivative. We report that altered GSH levels affect drug sensitivity to ART while the CQ response remains unchanged. This study provides new insights into the GSH involvement in the mechanism(s) of action of ART.

## Materials and Methods

### Mice and Parasites

Random-bred *Swiss albino* CD-1 female mice (Charles River Laboratories, Wilmington, MA, USA), 6–8 weeks old, weighting 20 to 35 g were used for the study. All mice procedures conducted at the AAALAC accredited UPR-School of Medicine were approved by the IACUC of the Medical Sciences Campus, University of Puerto Rico (Protocol numbers: 2480104; 2480106; 2480108, Animal Welfare Assurance # A3421-01). When an animal appeared to be in pain or disease the Veterinarian or Veterinary Technologist humanly euthanized the mouse by cervical dislocation or CO2 chamber following the American Veterinary Medical Association (AVMA) Guidelines for the Euthanasia of Animals. All work was done in strict accordance with the “Guide for the Care and Use of Laboratory Animals” (National-Research-Council, Current Edition) and regulations of the PHS Policy on Humane Care and Use of Laboratory Animals. Mice were maintained and housed according to NIH and AAALAC regulations and guidelines and were allowed to acclimatize for 1 week prior to the beginning of studies.

The *P*. *berghei* parasite lines used in this study were: ANKA 2.34 wild type reference line, *P*. *berghei* mutant clone *pbggcs-ko* [21], N clone (sensitive line) [22], RC line (selected under CQ pressure from the sensitive line) [22], and N/1100 (selected under MQ pressure) [23]. The Nclone, RC and N/1100 lines were derived from the K173 isolate while ANKA 2.34 clone was derived from the ANKA isolate [24].

For the RC or the N/1100 parasites, infections were started in a mouse by injecting an aliquot of parasites from liquid nitrogen stocks intraperitoneally. Mice were treated one day post-infection with CQ (for RC parasites, 60 mg/kg) or MFQ (for N/1100 parasites, 60 mg/kg) to maintain drug pressure. This treatment ensures that only resistant parasites will be further used in the study.

### Generation and genotyping of *pbggcs* overexpression parasites

**Fig 1:**
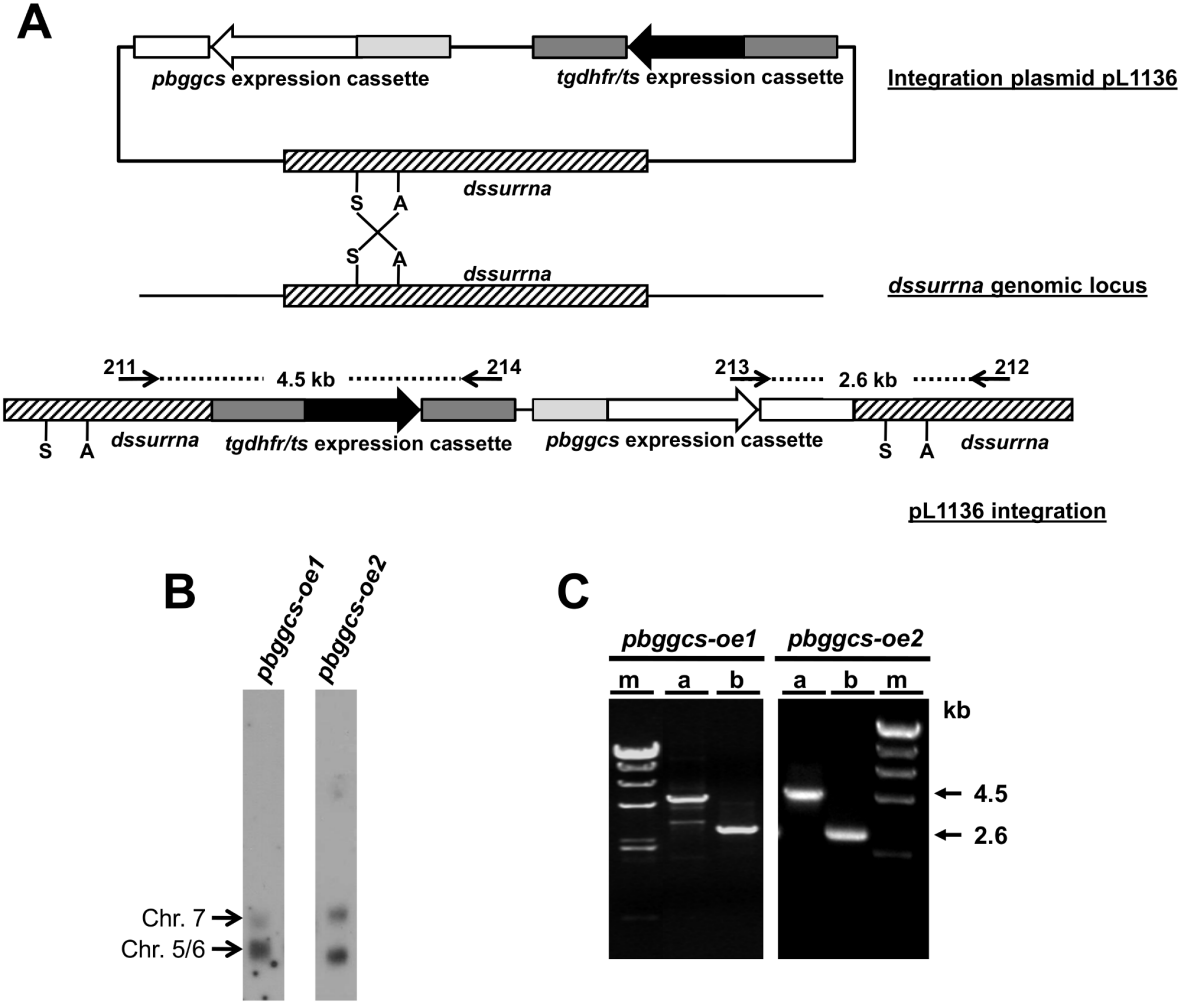
Generation of *pbggcs* overexpression mutants in *P*. *berghei*. **A.** Diagram of the *pbggcs* overexpression plasmid (Top), the *dssurrna* genomic locus (Middle) and the predicted integration event of the *pbggcs-oe* vector at the *dssurrna locus (chromosomes 5/6)* via single cross-over recombination (Bottom). Open arrows, *pbggcs* coding region; light grey boxes, *P*. *berghei* elongation factor *1a promoter;* white boxes, 3’UTR *pbggcs*; dark grey boxes, *P*. *berghei dhfr-ts* upstream and downstream regions; black arrows, *T*. *gondii dhfr-ts* coding region; hatched boxes, *dssurrna* DNA sequence*;* small arrows; positions of primer pairs for PCR analysis; dotted line, predicted size of PCR products; S, SacII; A, ApaI. **B)** Southern blot analysis of FIGE separated chromosomes of *pbggcs-oe* parasites. Correct integration of the *pbggcs-oe* plasmid to the *dssurrna* integration site was confirmed by incubating the chromosome blots with a *P*. *berghei* 3’UTR *dhfr-ts* specific probe that hybridizes to chromosomes 7 (endogenous *dhfr-ts*) and chromosome 5/6 (*dssurrna* integration site). **C)** PCR analysis confirming integration of the *pbggcs* over-expression constructs. Primers upstream and downstream from the endogenous *dssurrna* integration site (primers 211 and 212), to the *T*. *gondii dhfr-ts* selection cassette (primers 214) and to the integrated *pbggcs* sequence (primer 213) were used to verify integration into the *dssurrna* locus. Products of the predicted size (4.5 kb for primers 211/214 and 2.6kb for primers 212/213) were obtained for both clones. Lanes: m: 100 bp ladder (New England Biolabs), a: primers 211/214, b: primers 212/213.

The pL1136 vector containing the *Toxoplasma gondii dihydrofolate reductase—thymidylate synthase* (*tgdhfr*/*ts*) selectable marker was used as a backbone for the creation of a *pbggcs* over-expressing plasmid (Fig 1). The complete *pbggcs* ORF, including 465 bp of the 3’UTR, was amplified from ANKA 2.34 genomic DNA using primers 2562 (5’-CATGCCATGGATGGGTTTTCTAAAAATTGGAACTCC-3’; KpnI site is underlined) and 2563 (5’- CGGGGTACCTGGTGTGTATATACCAAACCGTTTC-3’; KpnI site is underlined), cloned into the TOPO TA vector (Invitrogene) and sequenced. The *pbggcs* coding sequence containing the 3’UTR was excised from the *pbggcs*-TOPO plasmid using the NcoI and the KpnI restriction enzymes and subsequently cloned into the pL0017 after removing the GFP coding sequence from the plasmid. The resulting pL1136 plasmid was linearized using the SacII restriction enzyme and transfected into *P*. *berghei* (ANKA 2.34) purified schizonts. Transfection, selection of transformed parasites with pyrimethamine, and cloning of *pbggcs*-oe parasites were carried out as previously described [25]. Clonal parasites (*pbggcs*-oe1; *pbggcs*-oe2) obtained by limiting dilution were analyzed for correct integration of the *pbggcs* over-expression plasmid into the c/d*-ssurrna* on chromosome 5/6 by Southern analysis of chromosomes separated by Field Inverted Gel Electrophoresis (FIGE). Chromosome Southern blots were hybridized with the *P*. *berghei* 3’UTR *dhfr-ts* specific probe (chromosome 7, endogenous *dhfr-ts* and chromosome 5/6,*dssurrna* integration site). Additionally, integration into the *dssurrna* locus was confirmed by PCR analysis of genomic DNA from the two mutants clones using specific primers for *pbggcs* 213 (5’-TGGGAAAAAGTTGTATCAATTC-3’), *pbdhfr/ts* 214 (5’-AGTCGGGAAACGTGTCGTG-3’) and *dssurrna* genes 211 (5’-CTTGCCAGTAGTCATATGCTTGT-3’) and 212 (5’-CTTCCGCAGGTTCACCA-3’).

Parasites with a disrupted *pbggcs* locus (*pbggcs*-ko1; *pbggcs*-ko2) were previously described in Vega-Rodríguez *et al*. (2009) [21].

### RNase protection assay

Expression of the *pbggcs* gene was analyzed by RNase Protection Assay (RPA) as previously described [26]. Briefly, Alpha-^32^P UTP labeled riboprobes for the *pbggcs* and the *β-tubulin* genes were synthesized *in vitro* by antisense transcription using the T7 RNA polymerase (Maxiscript SP6/T7 Kit, Ambion). RPA’s were performed using the RPAIII system (Ambion, Austin, Texas) according to the manufacturer’s instructions. Riboprobes were hybridized with total RNA from the *P*. *berghei pbggcs-oe* or wild type parasites overnight at 42°C. The probe-RNA hybrids were resolved on denaturing 6% acrylamide gels, which were subsequently exposed to autoradiography films. Autoradiograms were scanned and analyzed using Quantity One 1-D Analysis Software (Bio-Rad, v. 4.4). The density of the *pbggcs* signal was normalized to the density of the *β-tubulin* signal. Density ratios of the normalized *pbggcs* signals were subsequently normalized to ANKA to estimate mRNA expression levels in the *pbggcs*-*oe* parasites.

### Determination of GSH levels

Parasite GSH levels were determined by high-performance liquid chromatography (HPLC) as previously described [21, 27, 28]. Briefly, *P*. *berghei* infected blood was harvested from the donor mice with parasitemias between 5% and 15%. White blood cells were removed using a Whatman CF11 cellulose column [29]. The red blood cells (RBCs) were removed by lysis with saponin (0.15%) on ice, and free parasites resuspended at a concentration of 50X10^6^/100 ml in HPLC buffer (3.5 mM MgCl2, 110 mM KCl, 40 mM NaCl, 20 mM Hepes, 6 mM EDTA, pH 7.4) with protease inhibitors [30]. Parasites were lysed by three freeze/thaw cycles and parasite extracts were treated with an optimal concentration of dithioerythritol (12.5 mM) to reduce all the GSH derivatives [31]. Samples were resolved on a Hewlett Packard HP ODS Hypersil column and analyzed as Monobromobimane (MBrB) fluorescence derivatization in a Hewlett Packard 1050 Series HPLC.

### *In vivo* drug suppressive test

The Peters “*4 day suppressive test”* [32, 33] was carried out in *P*. *berghei* ANKA 2.34 wild type and the mutant parasites lacking (*pbggcs*-ko1; *pbggcs*-ko2) or overexpressing (*pbggcs*-oe1; *pbggcs*-oe2) the *pbggcs* gene. Two independent experiments were conducted for each parasite clone analyzed. CQ diphosphate salt and ART were obtained from Sigma-Aldrich. A 10 mg/ml stock solution was prepared in PBS for CQ and in 100% dimethyl sulfoxide (DMSO) for ART. Drugs were subsequently diluted in PBS to the appropriate dose for the drug assay. Five groups of mice (5 mice per group) were infected intravenously with 10X10^6^ parasites from each line and treated with CQ (intraperitoneal) and ART (subcutaneous) 1 hr post infection and then daily for three consecutive days with different drug doses (10 mg/kg, 3 mg/kg, 2 mg/kg, 1 mg/kg). Mice in the control group received vehicle alone (PBS or DMSO). On day 4 post-infection (5th day of assay), blood was collected and parasitemias determined from Diff Quick stained blood smears. A minimum of 350 RBCs were counted. Dose response curves and ED_50_ values were calculated after analysis with GraphPad Prism, version 4.03.

### ART (DHA) recrudescence assay

The 4 day suppressive test [32, 33] was modified to ascertain recrudescence after treatment with a 20 mg/kg dose of ART for four consecutive days. Mice (5 mice/group) were infected intravenously with either 10X10^6^ *P*. *berghei* ANKA 2.34 wild type, *pbggcs*-ko or the *pbggcs*-oe parasites. Mice were treated with intramuscular doses of 20 mg/kg of dihydroartemisinin (DHA) beginning 1 hr post infection, and daily for four consecutive days. Parasitemia was monitored daily after the fifth day of infection for up to 28 days by Diff Quick stained blood smears. Animal health was closely monitored and strict defined endpoints were followed. When an animal appeared to be in pain or disease the Veterinarian or Veterinary Technologist humanly euthanized the mouse by cervical dislocation or CO_2_ chamber following the American Veterinary Medical Association (AVMA) Guidelines for the Euthanasia of Animals. Euthanized mice are reported as mortality during recrudescence experiments.

## Results

### Overexpression of the *pbggcs* gene

**Fig 2:**
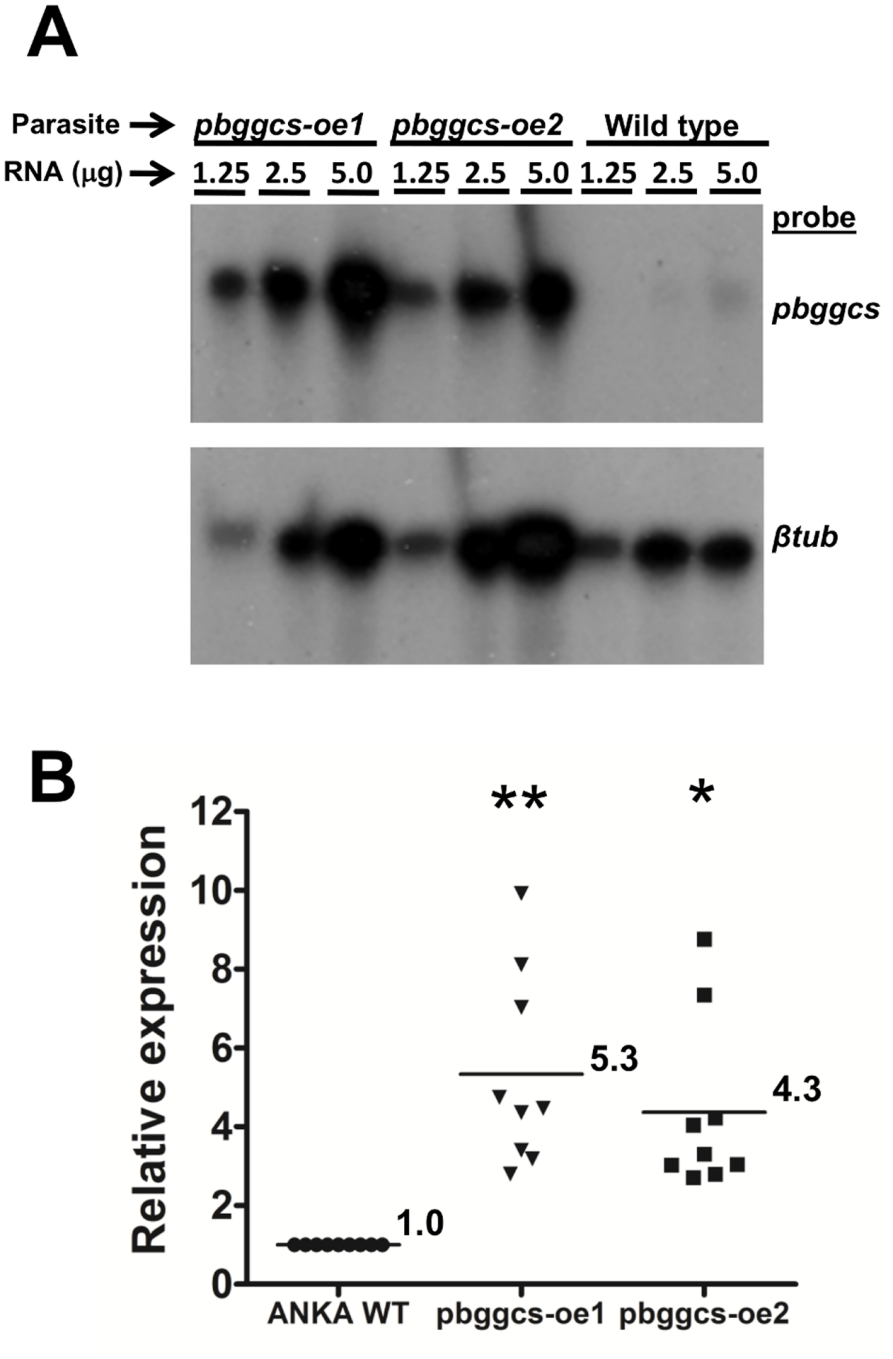
Overexpression of the *pbggcs* gene. **A)** Representative RPA showing *pbggcs* mRNA expression in wild type and *pbggcs-oe* parasites. Radiolabeled riboprobes for the *pbggcs* gene (top panel) and the *P*. *berghei* -tubulin gene (internal control, bottom panel) were used. **B)** Densitometric analysis of *pbggcs* expression by RPA. Densities of the *pbggcs* signals from each line were normalized to the density of the -tubulin signal. Normalized *pbggcs* signals from *pbggcs-oe* parasites were subsequently normalized to the signal of wild type. The horizontal line represents the mean relative expression of triplicate measurements from three independent experiments. Asterisks denote significant changes in *pbggcs* mRNA expression of *pbggcs-oe* parasites to wild type parasites as determined by a One-way ANOVA with Tukey’s Multiple Comparison Test (* = P<0.01, ** = P<0.001).

To assess the potential contribution of *Plasmodium* GSH levels to CQ and ART resistance, the single copy gene encoding the *P*. *berghei* γ-GCS was disrupted or overexpressed using standard genetic modification techniques. To overexpress the *pbggcs* gene, *P*. *berghei* parasites (ANKA 2.34) were transfected in two independent experiments with a DNA-construct designed to express the *pbggcs* gene driven by the *P*. *berghei* eukaryotic elongation factor 1a promoter (Fig 1A). Two parasite clones (*pbggcs-oe*1; *pbggcs-oe*2) from the two independent transfection experiments were isolated for further analysis. Integration of the construct into the genome of *pbggcs-oe*1 and *pbggcs-oe*2 parasites was confirmed by chromosome blots on field inverted gel electrophoresis (FIGE) separated chromosomes (Fig 1B) and by PCR analysis (Fig 1C). Densitometric analysis of the hybridization intensity of the *P*. *berghei* 3’UTR *dhfr-ts* specific probe on chromosome 5/6 (transgene insertion site) compared to the hybridization intensity on chromosome 7 (endogenous *pbdhfr/ts* locus, single copy gene) shows insertion of 3 and 2 copies of the *pbggcs* transgene on the *pbggcs-oe1* and the *pbggcs-oe2* parasites respectively (Fig 1B). Overexpression of *pbggcs* mRNA in blood stages of the *pbggcs-oe* parasites was demonstrated by RNase Protection Assay (Fig 2A and 2B). The *pbggcs* mRNA levels in *pbggcs-oe1* and *pbggcs-oe2* parasites were 5.3 (P<0.001) and 4.3 (P<0.01) times higher respectively relative to wild type parasites. These results demonstrate the successful over-expression of the *pbggcs* gene in the two mutant lines.

### Total GSH levels are increased in *pbggcs-oe* and in CQ and MQ *P*. *berghei* resistant parasites

**Fig 3:**
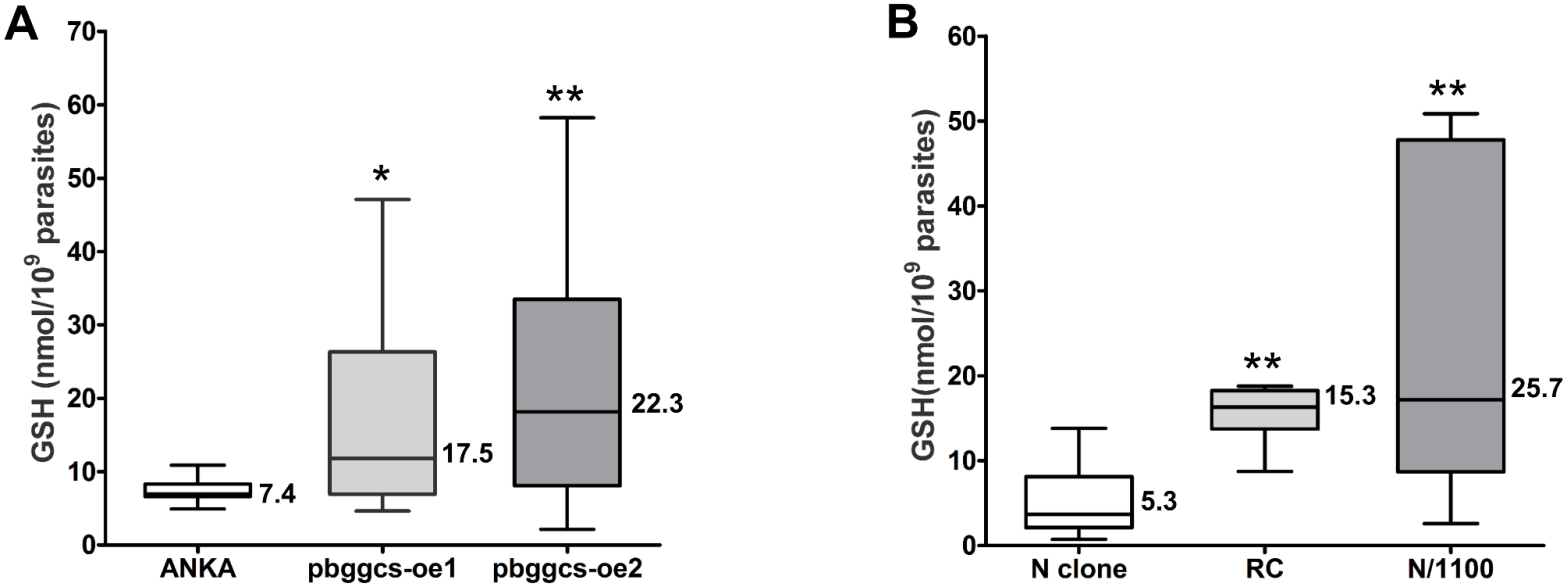
Total GSH levels in blood stages of *P*. *berghei* parasites. GSH levels were determined by HPLC in extracts of purified blood stage *P*. *berghei* parasites obtained from asynchronous infections of **(A)** ANKA wild type, *pbggcs-oe1* and *pbggcs-oe2*, **(B)** N clone (drug sensitive), RC (selected for CLQ resistant) and N/1100 (selected for MFQ resistant) parasites. GSH concentration was significantly increased in the *pbggcs-oe* parasites as compared to ANKA wild type. Similarly, the drug resistant lines RC and N/1100 displayed significantly higher GSH levels as compared to the sensitive one (N clone). Numbers on boxes represent the mean concentration of GSH. Asterisks denote significant changes in GSH concentration as determined by a One-way ANOVA with Tukey’s Multiple Comparison Test (* = P<0.05, ** = P<0.001).

To investigate whether or not overexpression of the *pbggcs* gene results in increased parasite GSH levels, total GSH was determined in *pbggcs*-oe and wild type parasites by HPLC. Total GSH levels were significantly higher in *pbggcs-oe1* (17.5 nmol/10^9^ parasites, SD ±15.2, P<0.05) and *pbggcs-oe2* (22.3 nmol/10^9^ parasites, SD ±19.3, P<0.001) parasites when compared to wild type (7.4 nmol/10^9^ parasites, SD ±1.7) (Fig 3A). These results show that overexpression of the *pbggcs* gene in *P*. *berghei* results in augmented GSH levels.

Increased levels of GSH were reported in CQ resistant *P*. *falciparum* (9)] and *P*. *berghei* [7, 11, 19]. Total GSH content was determined in the sensitive *P*. *berghei* N clone and the CQ-resistant RC [22] and MQ-resistant N/1100 [23] derived lines (Fig 3B). Significantly higher GSH levels were determined in the *P*. *berghei* CQ resistant RC (15.3 nmol/10^9^ parasites, SD ±3.1, P<0.001) and the MQ resistant N/1100 (25.7 nmol/10^9^ parasites, SD ±18.3, P<0.001) as compared to the sensitive N clone (5.3 nmol/10^9^ parasites, SD ±3.8). These results confirm previous reports which establish that in *P*. *berghei*, resistance to CQ is accompanied by an increase in total GSH levels [7–11].

### GSH levels do not alter *P*. *berghei* CQ or ART response in a 4-day suppressive assay

**Fig 4:**
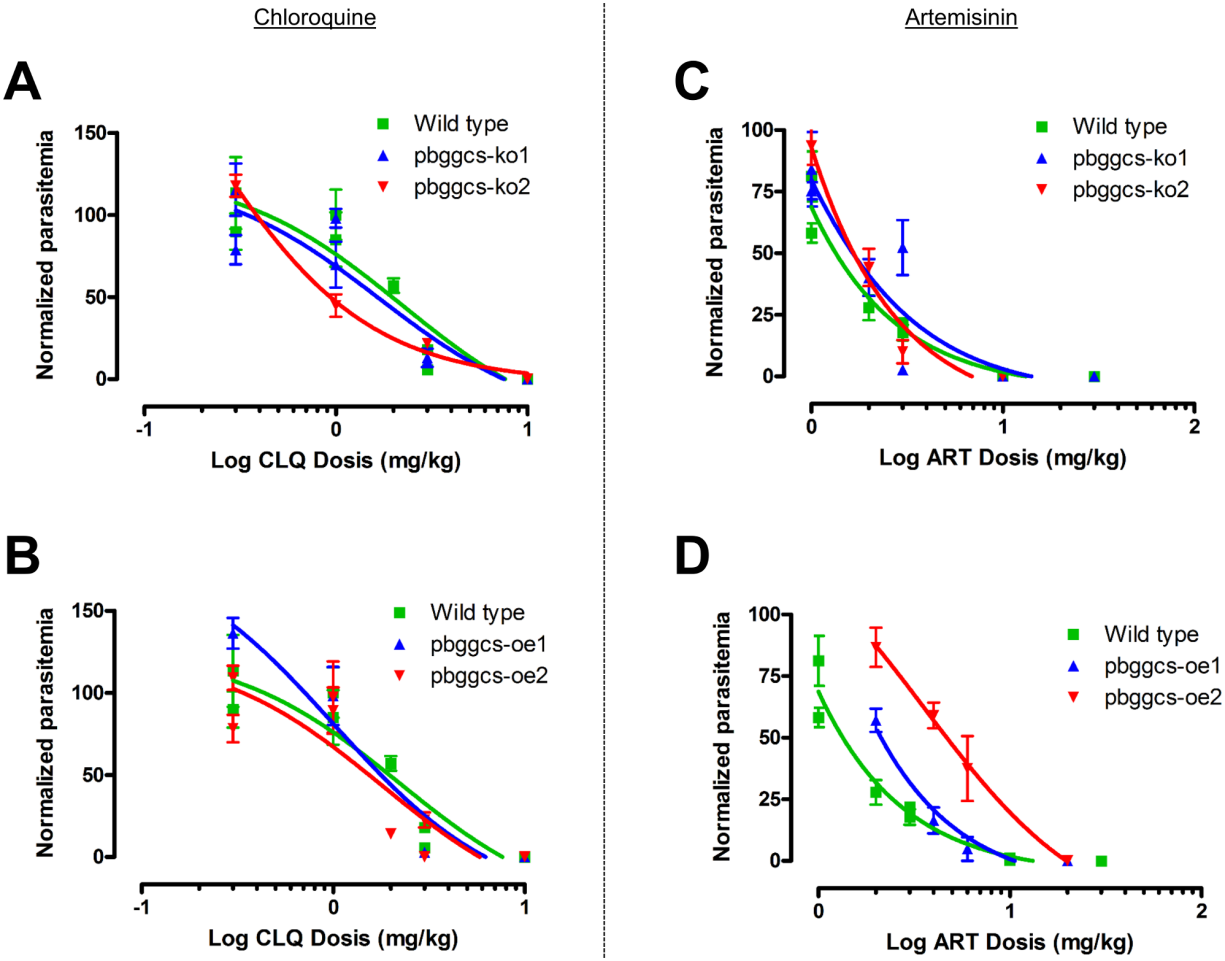
Drug response curves to CLQ and ART. Dose response curves show percent growth for each parasite clone relative to the untreated control on day 4 of the assay (5^th^ day post infection) versus drug concentration. The 4-day suppressive test was carried out for CLQ (A and C) and ART (B and D) on two independent *pbggcs-ko* (*pbggcs-ko1; pbggcs-ko2*) and *pbggcs*-oe (*pbggcs-oe1; pbggcs-oe2*) clones. To compare the drug responses from the mutant parasites, the parasitemia from each parasite line was normalized to the parasitemia of the untreated control. No significant differences in the EC_50_ values were observed between parasites with the *pbggcs* gene silenced or overexpressed as compared to wild type control. Bars represent standard deviation of the mean.

CQ and ART responses of *P*. *berghei* parasites displaying significantly high (*pbggcs-oe*) or significantly low (*pbggcs-ko*) [21] GSH levels were investigated. As determined by the 4-day suppressive assay, no major differences in CQ ED_50_ values were detected in the dose-response curves from parasites with low or high GSH levels when compared to wild type (Fig 4A and 4B). The CQ ED_50_ values for the *pbggcs-ko1* and *pbggcs-ko2* parasites were 1.7 mg/kg and 0.19 mg/kg respectively, while in the *pbggcs-oe1* and *pbggcs-oe2* were 1.01 mg/kg and 1.80 mg/kg respectively. The CQ ED_50_ value of wild type parasites was 2.21 mg/kg. Similarly, no significant differences in ART ED_50_ values were observed for *pbggcs*-*ko* and *pbggcs-oe* parasites when compared to wild type (Fig 4C and 4D). The ART ED_50_ values for the *pbggcs-ko1* and *pbggcs-ko2* parasites were 0.25 mg/kg and 0.015 mg/kg respectively, while in the *pbggcs-oe1* and *pbggcs-oe2* were 0.008 mg/kg and 3.58 mg/kg respectively. The ART ED_50_ value of wild type parasites was 0.016 mg/kg. These results show that either reducing or increasing the GSH levels in the *P*. *berghei* ANKA strain do not alter the response to CQ or ART in a 4-day drug suppressive test.

### *P*. *berghei pbggcs-ko* parasites failed to recover after ART treatment

**Fig 5:**
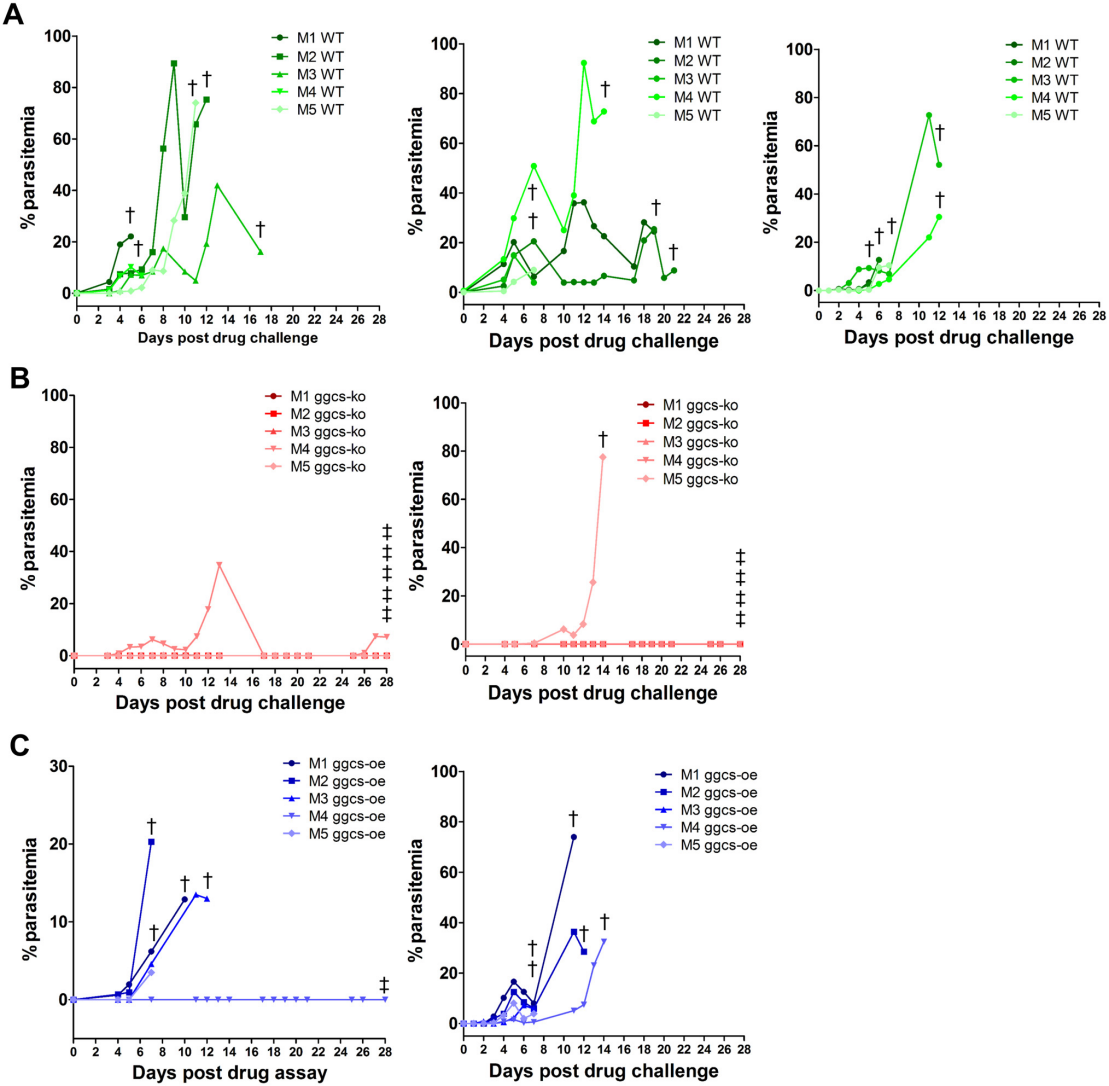
Recrudescence of *P*. *berghei pbggcs* deficient or overexpressing mutants after treatment with DHA. Mice infected with wild type (**A**), *pbggcs-ko* (**B**) or *pbggcs-oe* (**C**) parasites were treated with intramuscular doses of 20 mg/kg of DHA beginning 1 hr post infection, and daily for four consecutive days. After the 4^th^ DHA dose, every mouse on each group has undetectable parasite levels by microscopic examination. To determine recrudescence, the parasitemia was monitored in each mouse after the 4^th^ day of DHA treatment (day 0). 100% of the mice infected with wild type parasites (n = 15) and 90% of the mice infected with *pbggcs-oe* parasites (n = 10) presented recrudescence of the disease showing that these parasites were able to recover from the DHA treatment. Importantly, 80% of the mice (n = 10) infected with *pbggcs-ko* parasites remained parasite free throughout the duration of the assay suggesting that the knockout parasites did not recover from the DHA treatment. †, euthanized mouse due to malaria disease; ‡, euthanized parasite-free mouse. Each graph represents a biological replicate.

**Fig 6:**
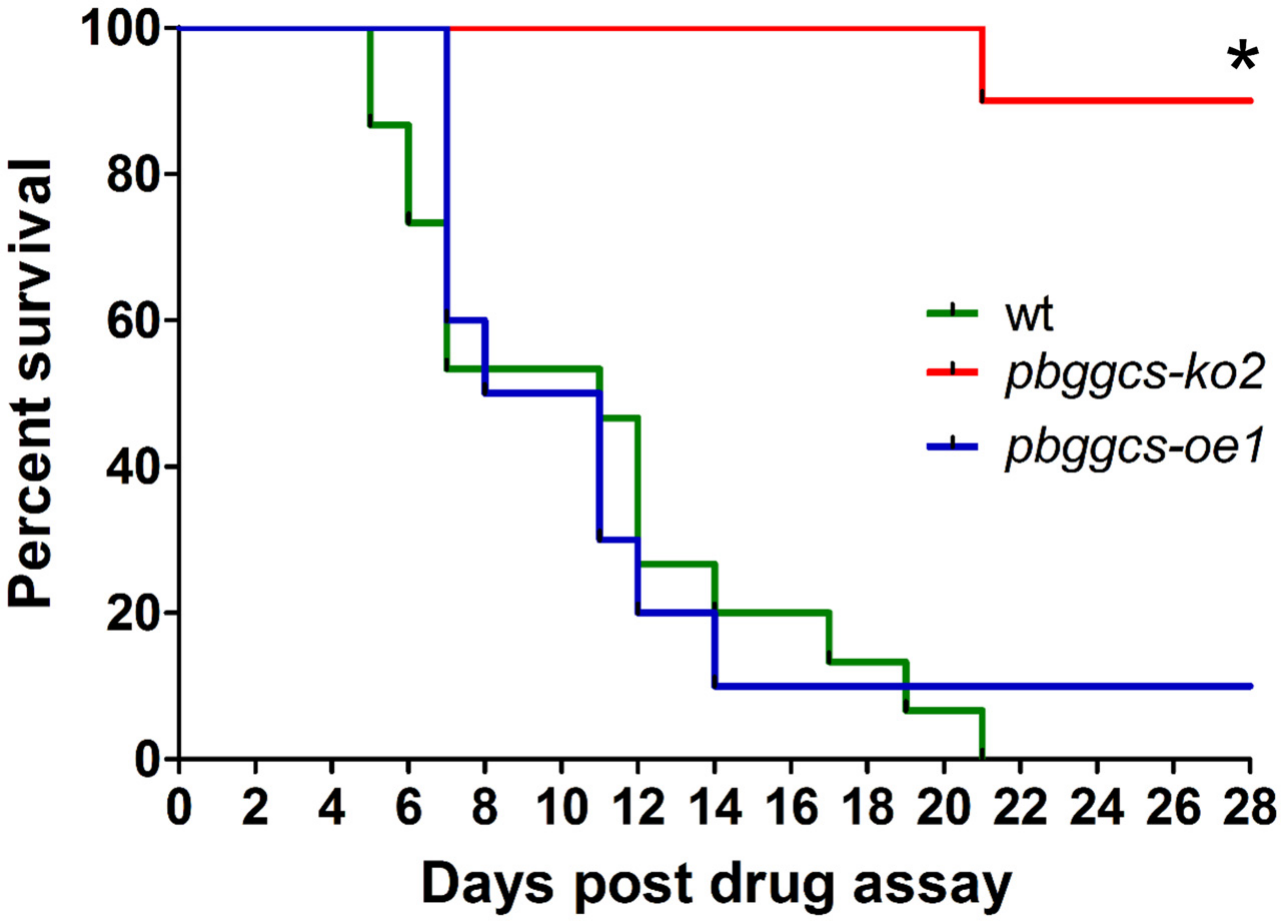
Kaplan-Meir survival curve of mice infected with *P*. *berghei* parasites lacking or overexpressing the *pbggcs* gene. Mice infected with wild type (green, n = 15), *pbggcs-ko* (red, n = 10) or *pbggcs-oe* parasites (blue, n = 10) were treated with intramuscular doses of 20 mg/kg of DHA beginning 1 hr post infection, and daily for four consecutive days. Mice survival was monitored after the 4^th^ day of DHA treatment (day 0). Mice mortality is defined as mice humanly euthanized due to distress caused by severe malaria. Survival of mice infected with wild type parasites was comparable to the survival of mice infected with *pbggcs-oe* parasites and rapidly declined after treatment with DHA. In contrast, 90% of the mice infected with *pbggcs-ko* parasites survived after the DHA treatment. Asterisk denote significant changes in survival as determined by a Log-rank (Mantel-Cox) Test (* = P<0.0001).

ART resistance is defined as an increase of parasite clearance time after ART treatment, or the presence of parasites on day 3 after treatment with recrudescence of the disease within 28 to 42 days [1]. The design of the 4 day test does not allows for detection of small changes in drug sensitivity as the one reported for ART resistance in field isolates [4, 5, 34]. A modification of the 4 day sensitivity test was employed in order to detect recrudescence after treatment with DHA, the active compound of ART and its derivatives. First, we determined that treatment of *P*. *berghei*-wild type infected mice with doses of 20 mg/kg DHA in a 4 day drug sensitivity assay results in microscopically undetectable levels of parasites at day 4 of the assay with reappearance of parasites circulating in the mouse peripheral blood within 2–3 days after treatment (Fig 5A). The *pbggcs-ko* and the *pbggcs*-oe parasites were subsequently analyzed for infection recrudescence after receiving a daily dose of 20 mg/kg DHA for four consecutive days. Parasites were undetectable after the fourth day of DHA treatment in all the parasite lines used (Fig 5). Mice infected with wild type parasites showed recrudescence between 2–3 days after treatment. In addition, nine out of the ten mice infected with *pbggcs-oe* parasites showed recrudescence (Fig 5A and 5B). Surprisingly, only two of the ten mice infected with the *pbggcs-ko* parasites showed recrudescence after ART treatment, one of which eventually cleared the infection (Fig 5B). The survival curves show that after treatment with DHA, survival of mice infected with *pbggcs-ko* was significantly higher (90%, P < 0.0001) than that of mice infected with wild type (0% survival) or *pbggcs-oe* (10% survival, P < 0.8021) parasites (Fig 6).

## Discussion

In this study, we demonstrate that overexpression of the *pbggcs* gene resulted in significantly increased GSH levels in blood stages. More importantly, we show that contrary to previous findings, low or high total GSH levels do not affect sensitivity to QC or ART in a 4-days drug suppressive test. Interestingly, recrudescence of parasites with low GSH levels (*pbggcs-ko*) after treatment with DHA is highly impaired, sustaining its role in the parasite’s response to ART.

*Plasmodium* resistance to CQ is mainly attributed to the acquisition of mutations in the *pfcrt* gene [35, 36]. In addition, mutations in the *pfmdr1* gene, encoding an ABC transporter, modulate levels of resistance. These mutations are associated with increased CQ efflux from the parasite’s digestive vacuole where CQ interferes with heme detoxification [35–38]. However, additional genes might be involved in conferring *Plasmodium* resistance to CQ [36, 39, 40]. Previous studies report that increased GSH levels are associated with *P*. *falciparum and P*. *berghei* resistance to CQ [7, 9, 19, 20, 41]. It is proposed that CQ can interfere with hemozoin polymerization by interacting with the m-oxo dimer form of oxidized heme [42–47]. Inhibition of hemozoin polymerization increases the parasites heme levels, which in turn increases oxidative stress resulting in damage to membranes and proteins [48, 49].

In this study, we analyzed the contribution of the antioxidant GSH on *Plasmodium* CQ resistance by using genetically transformed *P*. *berghei* lines with decreased (*pbggcs* knockout) or increased (*pbggcs* overexpression) levels of GSH. The response to CQ in both mutant lines was not affected when compared to wild type parasites suggesting that altered GSH levels do not modulate CQ drug resistance in *P*. *berghei*. The increased GSH levels previously reported in CQ resistant parasites could be the result of a parasite response to an oxidative stressed environment induced by CQ, including an increase in GSH production and/or changes in GSH transport. One of the most conclusive reports relating GSH to CQ resistance in *Plasmodium* is the reversion of CQ resistance by using BSO to deplete the GSH levels [7]. However, a study in *Trypanosoma brucei* suggested that BSO may have additional targets in the parasite besides the inhibition of the γ-GCS enzyme [50]. Supplementation of *T*. *brucei* with GSH rescued the lethal phenotype induced by the depletion of GSH after *ggcs* RNAi knockdown. However, supplementation with GSH did not complement the lethal phenotype seen by BSO treatment.

Alternatively, the increased GSH levels detected in CQ resistant *Plasmodium* parasites could be part of a resistance phenotype which in conjunction with other genes contributes to maintain the resistant phenotype [7, 9, 19, 41]. The genetic modifications (*pbggcs* knockout and knockin) resulting in altered GSH levels in *P*. *berghei* were done in the drug sensitive ANKA 2.34 strain of *P*. *berghei* which allows the analysis of drug resistance in parasites with high or low GSH levels under a similar genetic background. It is plausible that this strain does not possess the CQ resistant genetic background containing any of the CQ-resistance associated mutations present in the *pbcrt* gene. In support of this hypothesis, Patzewitz *et al*. (2013) reported that the transporter encoded by the *P*. *falciparum* CQ resistant *pfcrt* allele was able to transport GSH more efficiently than the CQ sensitive allele [20]. They suggested that an increase of GSH import into the digestive vacuole, presumably mediated by the CQ resistant PfCRT, and not the augmented levels of total parasite GSH, could cause an increase in parasite resistance to CQ [20]. It is conceivable that the sensitivity of the *pbggcs-ko* (low GSH) and the *pbggcs-oe* (high GSH) parasites did not change to that of wild type parasites because of the absence of the CQ resistance genotype, such as *crt* mutations. In this report significantly increased GSH levels were demonstrated in the *P*. *berghei* CQ resistant RC line and in the MQ and CQ resistant N/1100 line when compared to the sensitive N clone. Disruption of the *pbggcs* gene in the CQ resistant RC line to reduce the GSH levels could help to prove the above hypothesis, as this line may possess mutated *crt* and/or *mdr* genes.

When *pbggcs-ko* or *pbggcs-oe* parasites were tested for ART response in a 4 day sensitivity assay, both mutant parasites presented a drug response similar to the wild type control. However, when analyzed in a recrudescence assay, the *pbggcs-ko* parasites did not recover from the treatment as evidenced by the lack of infection after day five. These results show that reduced levels of the antioxidant GSH renders *P*. *berghei* parasites more sensitive to ART treatment. This is compatible with the ART resistance phenotype detected in *P*. *falciparum* from Southeast Asia which is characterized by the presence of parasites on day 3 after ART treatment with a concomitant recrudescence of the disease [1].

The antimalarial activity of ART is thought to result from an altered redox balance in the parasite caused by this endoperoxide-containing drug. ART induces the autoxidation of flavin cofactors, including FADH_2_, which is required by the GSH reductase enzyme for the reduction of GSSG to GSH [51]. GSH can also form adducts with an ART derived C-centered primary radical [16]. In addition, hemoglobin degradation by the parasite is required for both the increased levels of ROS and the antimalarial activity of ART [15]. Malaria parasites are rich in hemin, which results from hemoglobin degradation. ART interacts with hemin to produce ROS, resulting in cellular damage [13]. In support of these findings, Paitayatat *et al*. (1997) reported that the ART binding affinity to hemin correlates with the ART anti-plasmodial activity [14]. The above mentioned reactions will reduce the intracellular pools of reduced GSH resulting in an increase of ROS-induced damage. Our previous report show that *P*. *berghei* blood stages survive with very low GSH levels [21]. However, this reduced pool of GSH might render the parasite even more sensitive to the oxidative stress induced by ART. In addition, ART can react with GSH *in vitro* and it is proposed that glutathione S-transferase might be involved in the metabolism of ART [52].

Recently, mutations in the parasites PF3D7_1343700 kelch propeller domain (K13-propeller) protein were associated with resistance to artemisinin in *P*. *falciparum* laboratory strains and field isolates [6, 53, 54]. Some of these mutations are highly prevalent among *P*. *falciparum* isolates from patients that show a delayed clearance of parasites after ART treatment [6, 53]. Removal of these mutations from ART resistant isolates reduced the ART survival rate of the parasite [54]. In addition, introduction of the K13-propeller mutations into the ART sensitive allele increased the ART resistance levels to those observed in *P*. *falciparum* field isolates carrying this mutation [54, 55]. The *P*. *falciparum* K13-propeller has homology with the human KEAP1, a kelch domain-containing protein [53]. KEAP1 is a repressor of the transcription factor Nrf2, which in turn induces the expression of cytoprotective and antioxidant enzymes, including some of the enzymes of the GSH metabolism like γ-GCS and GSH S-transferase [56]. Based on homology to other kelch domain-containing proteins, it is possible that the mutations associated with ART resistance in the *P*. *falciparum* K13-propeller could destabilize the kelch domain scaffold and alter the protein function [53].

In summary, we report here that altered GSH levels do not change *P*. *berghei* sensitivity to CQ. However, reduction of GSH levels renders *P*. *berghei* parasites more sensitive to clearance by ART treatment. These results suggest that ART resistant parasites could be using antioxidant molecules like GSH to reduce the pro-oxidant effects of ART resulting in an increase of tolerance to the drug. A better understanding of the potential contribution of GSH to the modulation of ART resistance could help improve current strategies using ART or ART-based combination therapies to control malaria.
